# Complete genome sequence of *Escherichia coli* phage Eryne

**DOI:** 10.1128/mra.01218-24

**Published:** 2025-03-05

**Authors:** Valentin Druelle, Alexander Harms

**Affiliations:** 1 Biozentrum, University of Basel 27209 , Basel, Switzerland; 2 Institute of Food, Nutrition and Health, ETH Zurich, Zurich, Switzerland; University of Maryland School of Medicine, Baltimore, Maryland, USA

**Keywords:** bacteriophage, *Justusliebigvirus*, tailspike, *Escherichia coli*

## Abstract

Bacteriophage Eryne is a new virus infecting clinical and laboratory strains of *Escherichia coli* that targets surface glycans. We report the 145,026 bp genome of phage Eryne and show that it belongs to the genus *Justusliebigvirus* of the *Stephanstirmvirinae,* known for their multivalent host recognition.

## ANNOUNCEMENT

**Fig 1:**
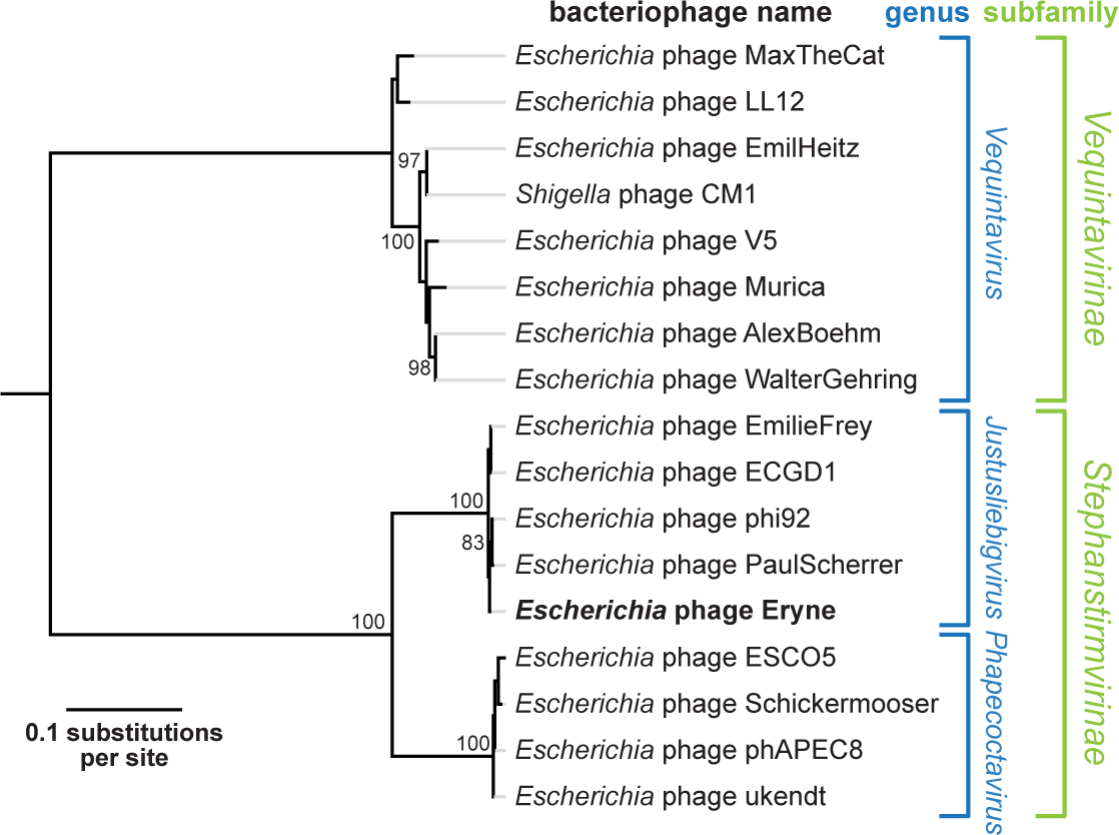
Major capsid protein gene phylogeny of phage Eryne. The major capsid protein genes of phages belonging to *Stephanstirmvirinae* genus *Justusliebigvirus* and relatives were identified as best hits of the Eryne ortholog by BLASTn searches against the non-redundant nucleotide database of NCBI GenBank (release 262) and aligned using MAFFT (version 7.490) implemented in Geneious Prime 2024.0.2 with FFT-NS-1 algorithm, 200PAM/*k* = 2 scoring matrix, gap open penalty 1.53, and offset value 0.123 (default settings; alignment length 1,036 nt) (14). Subsequently, we calculated a maximum likelihood phylogeny based on this alignment using PhyML (version 3.3.20180621) (15) implemented in Geneious Prime 2024.0.2 with the HKY85 substitution model and 100 bootstraps. The phylogeny was rooted between *Vequintavirinae* and the other phages. Bootstrap values are shown if >70/100. Phage Eryne is highlighted in bold.

Phages of genus *Justusliebigvirus* (phi92-like phages) and their relatives encode several co-expressed tailspikes and tail fibers targeting diverse host receptors, which enable remarkably broad host recognition analogous to a “Swiss Army knife” and hold great potential for biotechnology (1–3). Here, we report the genome of phage Eryne, a new *Justusliebigvirus* phage. Eryne was isolated in 2020 from wastewater from the local sewage treatment plant of the city of Basel (ARA Basel, 47°34′53.6″N 7°36′00.9″E) by plating on a lawn of *Escherichia coli* strain UTI89 grown in LB soft agar at 37°C as described previously (2). Plaques obtained with this uropathogenic model strain were tested for growth on the *E. coli* K-12 MG1655 ΔRM with and without restored expression of O-antigen glycans (*wbbL*(*+*) genotype [2]). Phage Eryne grew on both strains, forming clear plaques of 1–2 mm diameter on *E. coli* K-12 with O-antigen and very small, turbid plaques without O-antigen expression. These results suggest that Eryne encodes a tailspike recognizing the O16-type O-antigen of *E. coli* K-12 but can only poorly recognize the NGR glycan that seems to be targeted by *Justusliebigvirus* phages on K-12 strains in its absence (4). The phage was passaged three times to ensure a pure, clonal stock before generating a high-titer lysate using double-agar overlays. Briefly, an LB soft agar with the *E. coli* K-12 MG1655 ΔRM *wbbL*(*+*) host was infected with Eryne for confluent lysis and grown for ca. 16 h at 37°C before being scraped off the plate into ca. 15 mL of SM buffer. This suspension was heavily vortexed and cleared from debris by centrifugation at 8,000 *g* for 5 minutes to generate the phage stock. Subsequently, the genomic DNA of Eryne was extracted from 1 mL of high-titer stock using the Norgen Biotek Phage DNA Isolation Kit. For sequencing at SeqCenter (Pittsburgh, USA), sample libraries were prepared using the protocol of Baym and colleagues (5) with dual 8 bp indices and sequenced on an Illumina NextSeq 550 producing 2 × 151 bp reads. The data were demultiplexed and adapters were removed using bcl2fastq (version 2.20.0.422; Illumina Inc., San Diego, USA) without additional trimming or quality control at this stage (91% of base pairs had a quality score of Q30). We then used Unicycler version 0.5.0 in Illumina-only mode to assemble 100,000 reads randomly subsampled by Seqtk-1.3 (6) from the total of 2,386,786 reads after automated quality control with FastQC (7) and obtained a circular assembly of 145,026 bp with a GC content of 37.4% and a mean coverage of 165× (8). The genome of Eryne was annotated using Pharokka version 1.7.3 and Phold version 0.2.0 with standard settings after which it was linearized with the 5′ end at the start codon of the large terminase gene (9, 10). Using BLAST (version 2.16.0) of the whole genome of Eryne against the non-redundant nucleotide database of NCBI GenBank (release 262) (11, 12), we determined that this phage belongs to the genus *Justusliebigvirus* of the *Stephanstirmvirinae* subfamily by showing >98% identity over >98% of the query sequence for known representatives like PaulScherrer (cutoff: 70% identity across genome length [13]). Phylogenetic analyses confirmed the identity of Eryne as a *Justusliebigvirus* (Fig. 1).

## Data Availability

This whole genome shotgun project has been deposited under accession number PQ529420 with the version described in this paper being the first version. Sequencing reads have been deposited in the sequencing read archive (SRA) under accession number SRR31253914.
